# Glucocorticoids injure the airway epithelial barrier via endoplasmic reticulum stress-related apoptosis in asthma

**DOI:** 10.3389/fphar.2026.1771751

**Published:** 2026-05-08

**Authors:** Hao You, Wenjing Zou, Kunpeng Wang, Tangqiaochu Gan, Jie Hu, Wen Tan, Ting Wang, Luo Ren, Gang Geng, Zhou Fu, Chao Niu

**Affiliations:** 1 Department of Respiratory Medicine Children’s Hospital of Chongqing Medical University, National Clinical Research Center for Children and Adolescents’ Health and Diseases, Ministry of Education Key Laboratory of Child Development and Disorders, Chongqing Municipal Health Commission Key Laboratory of Children’s Vital Organ Development and Diseases, Chongqing, China; 2 Chongqing Medical University, Chongqing, China; 3 Department of Pediatric Respiratory Medicine, Chengdu Women’s and Children’s Central Hospital, School of Medicine, University of Electronic Science and Technology of China, Chengdu, China; 4 Chongqing Key Laboratory of Child Rare Diseases in Infection and Immunity, Chongqing, China, Key Laboratory of Children’s Important Organ Development and Diseases of Chongqing Municipal Health Commission, Chongqing, China

**Keywords:** airway epithelium, apoptosis, asthma, endoplasmic reticulum stress, glucocorticoids

## Abstract

**Introduction:**

Glucocorticoids serve as the first-line clinical treatment for asthma; however, 5%–10% of asthma patients respond poorly to glucocorticoids, and their underlying mechanism remains unclear. We investigated whether glucocorticoids can induce airway epithelial endoplasmic reticulum (ER) stress-related apoptosis to decrease their efficacy in treating asthma.

**Methods:**

We established a BALB/c mouse model of OVA-induced asthma and treated the mice with the glucocorticoid dexamethasone. H&E staining, bronchoalveolar lavage assays and lung function tests were performed. We also treated 16HBE cells with dexamethasone and ER stress inhibitors (4μ8C and melatonin). The viability and apoptosis of 16HBE cells were measured by the CCK-8 and TUNEL assays, respectively. The levels of ER stress markers, including ATF6, ATF4, CHOP and XBP1s, were measured by qPCR and Western blotting.

**Results:**

Dexamethasone decreased airway inflammatory infiltration and Penh values in asthma model mice but seemed to exacerbate airway epithelium defects in the trachea. qPCR revealed that dexamethasone increased ATF4, CHOP and XBP1 mRNA levels in the lungs of asthma model mice. Moreover, dexamethasone increased apoptosis, as determined by TUNEL assays, and upregulated ATF6, ATF4, CHOP, and XBP1s expression, as determined by Western blotting, in 16HBE cells. The ER stress inhibitors 4μ8C and melatonin decreased the negative effects of dexamethasone on 16HBE cells.

**Discussion:**

Glucocorticoids may injure the airway epithelial barrier via ER stress-related apoptosis in asthma. ER stress inhibition may decrease the negative effects of glucocorticoids on the airway epithelial barrier and consequently improve the efficacy of glucocorticoids in asthma. This study proposes a novel potential explanation for glucocorticoid resistance and justify further clinical investigation.

## 1 Introduction

Asthma is characterized by chronic airway inflammation, airway hyperresponsiveness (AHR), and airway remodeling; it is a heterogeneous disorder that manifests as dyspnea, wheezing, and cough (Pavord et al., 2018). Asthma is the most prevalent chronic respiratory disease in children, with approximately 334 million individuals being affected worldwide (Savin et al., 2023). Glucocorticoids (GCs) are currently the first-line treatment for the clinical management of asthma; GCs provide effective asthma control and improve lung function in most asthma patients; however, 5%–10% of asthma patients respond poorly to GCs, and the underlying mechanism of action of GCs is unclear (Pj, 2022; Kim and Agrawal, 2025).

The airway epithelium is the first defensive barrier in the host airway. Almost all patients with asthma exhibit airway epithelial injury and display reduced barrier function (Gay et al., 2024; Raby et al., 2023). If airway epithelial integrity is impaired, inhaled toxins and pathogens can enter the body much more easily, possibly causing an asthma attack or even chronic airway inflammation, airway hyperresponsiveness and mucous hypersecretion. The airway epithelial barrier of asthma patients is subjected to various stressors, such as the accumulation of airway mucus and infectious and noninfectious inflammation, and we believe that GCs may increase stress on the airway epithelial barrier, further affecting the efficacy of glucocorticoids in treating asthma. Stress on the airway epithelial barrier manifests as endoplasmic reticulum (ER) stress.

ER stress is a cellular response triggered by the accumulation of misfolded or unfolded proteins under various physiological and pathological conditions, leading to a series of signaling cascades, such as the unfolded protein response (UPR) (Siwecka et al., 2019). Studies have indicated that ER stress, as the main response to organelle stress (Osorio et al., 2013), plays a pivotal role in regulating apoptotic pathways (Felismino and Coimbra, 2023; Costa-Mattioli and Walter, 2020; Eltokhy et al., 2022). The aim of this study was to investigate whether glucocorticoids may induce airway epithelial endoplasmic reticulum (ER) stress-related apoptosis and to decrease their efficacy in treating asthma. These findings are expected to provide a theoretical foundation and new insights for improving therapeutic efficacy in patients with asthma.

## 2 Materials and methods

### 2.1 Mice and treatment

Six-to eight-week-old female BALB/c mice were purchased from the Experimental Animal Center of Chongqing Medical University. The mice were housed under specific pathogen-free conditions on a 12-h:12-h light/dark cycle and fed an OVA-free diet. A total of 72 animals were used in these experiments (nine per group). The study was approved by the Ethics Committee of Chongqing Medical University (reference number: 2012023).

A mouse model of OVA-induced asthma was established according to previously published methods with minor modifications (Oh et al., 2002; Tian et al., 2008; Burr et al., 2021). Briefly, 6–8-week-old mice were sensitized on days 0, 7, and 14 via intraperitoneal and subcutaneous injections of 200 μL of an emulsion containing 100 μg of OVA and 2 mg of Al(OH)_3_. From days 21–30, airway inflammation was provoked by daily 30-min aerosol challenges with 1% OVA. On the final 3 days (days 28–30), the designated treatment groups received intraperitoneal injections of either 1 mg/kg or 5 mg/kg dexamethasone 30 min before challenge, whereas the asthmatic control group was administered PBS. At the end of the experiment, all mice were sacrificed by means of cervical dislocation.

### 2.2 Reagents and antibodies

The following materials and reagents were used in this study: 4μ8C (HY-19707, MCE), melatonin (HY-B0075, MCE), dexamethasone (Meilunbio, MB1434-2), a CCK-8 assay kit (Apexbio, K1018) and a TUNEL assay kit (Meilunbio, MA0223).

Antibodies against EGFR (Cell Signaling, 4267), BAX (CST, 5023), BCL2 (CST, 4223), XBP1s (Proteintech, 24868-1-AP), GAPDH (Proteintech, 10494-I-AP), ATF4 (Proteintech, 60035-1-Ig), ATF6 (Proteintech, 66563-1-Ig) and CHOP (Proteintech, 66741-1-Ig) were used for Western blotting.

### 2.3 Noninvasive measurement of enhanced pause

Penh was measured within 24 h after the final OVA airway challenge. The mice were placed into a whole-body plethysmograph (EMKA Technologies, Paris, France) and exposed to increasing concentrations (0, 3.125, 6.25, 12.5, 25 and 50 mg/mL) of acetyl-β-methylcholine chloride (methylcholine; Sigma-Aldrich, Saint Louis, MO, USA). Penh was subsequently recorded according to previously described methods (Niu et al., 2019; Wang et al., 2017). The airflow obstruction index is expressed as Penh and is related to airway responsiveness (Niu et al., 2019; Dong et al., 2015; Shen et al., 2025).

### 2.4 Broncho-alveolar lavage fluid assays

Following sacrifice, the trachea of each mouse was cannulated, and the BAL fluid was collected by six injections of 0.5 mL of PBS into the lungs.

Cells were collected from the BAL fluid by centrifugation, after which red blood lysis buffer was added to remove the red blood cells, and the cells were counted by microscopy using a cell hemocytometer. Differential cell counts were performed based on standard morphological and staining characteristics of at least 200 cells per sample (Yang et al., 2024). The remaining BAL fluid was centrifuged, and the collected supernatant was analyzed for cytokines.

### 2.5 Cytokine analysis

Commercially available enzyme-linked immunosorbent assay kits for IL-5 (≥8 pg/mL; 4 A Biotech, Beijing, China) and IFN-γ (≥8 pg/mL; Neobioscience, Shenzhen, China) were used to measure the concentrations of these cytokines in the BAL fluid.

### 2.6 Histological staining

Immediately following euthanasia, the tissues were harvested and fixed in 4% buffered formaldehyde, dehydrated in a graded alcohol series, cleared with dimethylbenzene, and embedded in paraffin. The paraffin blocks were serially sectioned at a thickness of 4 μM and stained with hematoxylin and eosin (H&E) using standard techniques.

### 2.7 Real-time quantitative PCR

Total RNA was isolated from mouse lung tissue, and cDNA synthesis was performed with a PrimeScript RT Reagent Kit (Takara, Otsu, Japan). Real-time quantitative PCR (qPCR) was performed using standard techniques. For each sample, GAPDH were used as internal controls. The sequences (5′>3′) of the primers were 5′-TGT​ATT​ACG​CCT​CCC​CTG​GA-3′ (forward) and 5′-GCG​CAC​TTC​CTG​TTC​TCT​CT-3′ (reverse) for ATF6, 5′-GGA​GGC​TTA​CAC​AAT​GGC​CT-3′’ (forward) and 5′-TCT​GTC​CCG​GAA​AAG​GCA​TC-3′ (reverse) for ATF4, 5′-CTT​TTG​CCC​AAG​CTT​CCC​AC-3′ (forward) and 5′-GAT​AGG​CTT​TCT​CCG​GGC​TC-3′ (reverse) for CHOP, and 5′-TTA​GTG​ACG​GAA​CCA​CAC​GG-3′ (forward) and 5′-GAA​CAC​AAA​CCT​GCA​GCC​AG-3′ (reverse) for XBP1s.

### 2.8 Cell culture and grouping

The 16HBE bronchial epithelial cells were obtained from retained cell stocks preserved in liquid nitrogen by the research group and were cultured with complete medium (10% fetal bovine serum +90% basal medium) in a cell incubator containing 5% CO_2_ at 95% humidity and 37 °C. The cells were divided into the following groups: the control group, dex groups and ER stress inhibitor groups. The dex groups were treated with 5–20 μM dexamethasone, and statistically significant differences were observed among these groups (Supplementary Figure S1A); the ER stress inhibitor groups were treated with both 10 μM dexamethasone and an ER stress inhibitor such as 1 μM 4μ8C or melatonin, which was found to be noncytotoxic to the cells (Supplementary Figures S1B,C). The control group was treated with an equal volume of phosphate-buffered saline (PBS).

Unless otherwise specified, the duration of drug exposure was 24 h for all experiments.

### 2.9 Cell viability assay

16HBE cells were seeded in 96-well culture plates and subsequently grouped and treated as *2.8* described. To assess cell viability, we used a CCK-8 kit according to the manufacturer’s instructions and measured the absorbance at 24, 36, and 48 h after the initiation of drug intervention; a higher absorbance indicated a greater number of viable cells and consequently greater cell viability.

### 2.10 TUNEL assay

16HBE cells were seeded in 35 mm dishes and then grouped and treated as *2.8* described. Apoptosis was subsequently detected using a TUNEL assay kit according to the manufacturer’s instructions. Apoptosis was subsequently examined under a laser scanning confocal microscope.

### 2.11 Western blotting

Total cell lysates were obtained using a Total Protein Extraction Kit (KeyGen Biotech) according to the manufacturer’s instructions. Afterward, the proteins were transferred to PVDF membranes by SDS-PAGE and probed with various primary antibodies and fluorophore-labeled secondary antibodies. Finally, the blots were visualized using a fluorescence imaging system.

### 2.12 Statistical analyses

Statistical analysis was performed using GraphPad Prism 6.0, and the data are presented as $\bar{x}$ ± s. All *in vitro* experiments were performed in at least three replicates. For animal experiments, all measurements were conducted in a blinded manner. The two sets of data were analyzed using the t-test, while multiple groups were analyzed using one-way ANOVA (when assumptions of normality and homogeneity of variance were met) or the Kruskal–Wallis test (otherwise). *P < 0.05* was considered to indicate statistical significance.

## 3 Results

### 3.1 Successful establishment of a mouse model of ovalbumin-induced asthma

A BALB/c mouse model of asthma was established as shown in the schematic (Supplementary Figure S2A). The successful establishment of a mouse model of OVA-induced asthma was confirmed through hematoxylin and eosin (H&E) staining, along with analysis of the levels of representative airway cytokines, the number of shed airway epithelial cells and inflammatory cells in the BALF and Penh values (Supplementary Figures S2B–E).

### 3.2 The GC dexamethasone alleviated airway inflammation but failed to alleviate injury to the airway epithelial barrier in a mouse model of asthma

**FIGURE 1:**
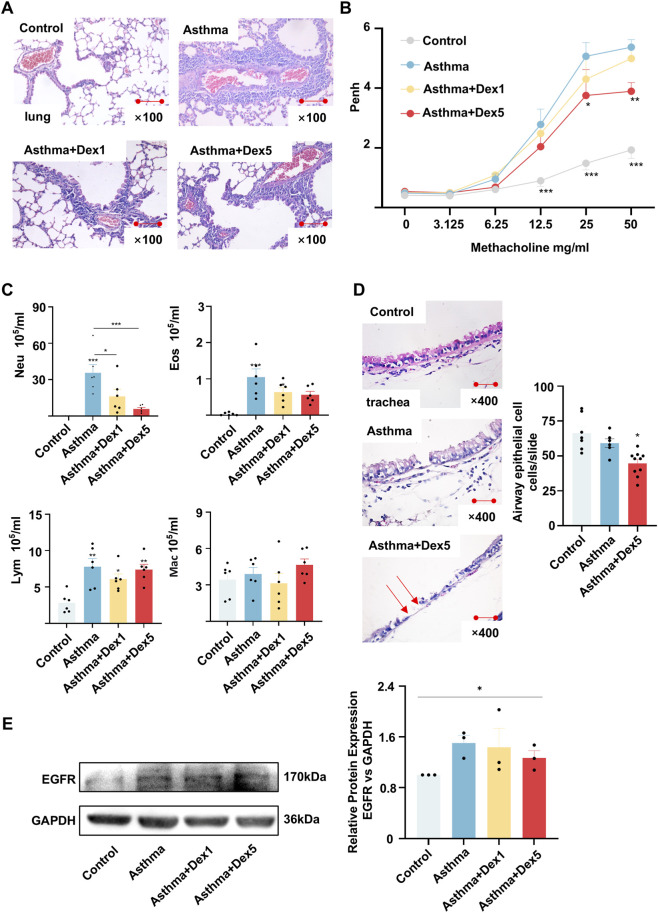
The glucocorticoid dexamethasone alleviated airway inflammation and airway hyperresponsiveness but injured the airway epithelial barrier in a BALB/c mouse asthma model. **(A)** Representative hematoxylin and eosin (H&E)-stained lung tissue sections. The number of inflammatory cells around the airways was increased in a BALB/c mouse asthma model, and dexamethasone alleviated airway inflammatory infiltration. **(C)** Total inflammatory cell counts in broncho-alveolar lavage fluid (BALF). In a BALB/c mouse asthma model, the number of airway inflammatory cells in the BALF was increased; however, dexamethasone decreased the number of these cells. **(B)** Airway hyperresponsiveness assessed by enhanced pause (Penh) values. The Penh value was increased in the BALB/c mouse asthma model, whereas dexamethasone decreased this value. **(D)** Representative hematoxylin and eosin (H&E)-stained trachea tissue sections. In a BALB/c mouse asthma model, the airway epithelial barrier was injured, as well as reduction of airway epithelial cell counts, whereas dexamethasone failed to repair the epithelial barrier. The arrows indicate aggravated epithelial injury. *P < 0.05, **P < 0.01, ***P < 0.001 using one-way ANOVA. **(E)** Protein expression levels of EGFR, as determined by Western blotting and quantitative analysis, *P < 0.05 using Kruskal-Wallis test.

To observe the effects of GCs on the airway epithelial barrier, we generated a mouse model of OVA-induced asthma and treated the mice with dexamethasone, as shown in the schematic (Supplementary Figure S3). Compared with the asthma group, the asthma + Dex1 and asthma + Dex5 group exhibited significant decreases in airway inflammation, as indicated by H&E staining (Figure 1A), Penh values (Figure 1B) and the number of inflammatory cells in the broncho-alveolar lavage fluid (BALF) (Figure 1C). Paradoxically, the defects were aggravated in the tracheal epithelium of the OVA-challenged mice treated with dexamethasone (Figure 1D). Additionally, we performed Western blotting to measure the protein expression level of EGFR, a marker of epithelial injury, in mouse lung tissues. The results showed that dexamethasone failed to reduce epithelial impairment in asthmatic airways (Figure 1E).

### 3.3 The GC dexamethasone may have decreased cell viability and promoted apoptosis in airway epithelial 16HBE cells

To assess the negative effect of dexamethasone on airway epithelial cells, we performed multiple experiments using 16HBE cells, including analysis of cell morphology under microscopy, the CCK-8 assays, Western blot analysis of BAX/BCL-2 expression, and TUNEL assays, to examine cell viability and apoptosis.

**FIGURE 2:**
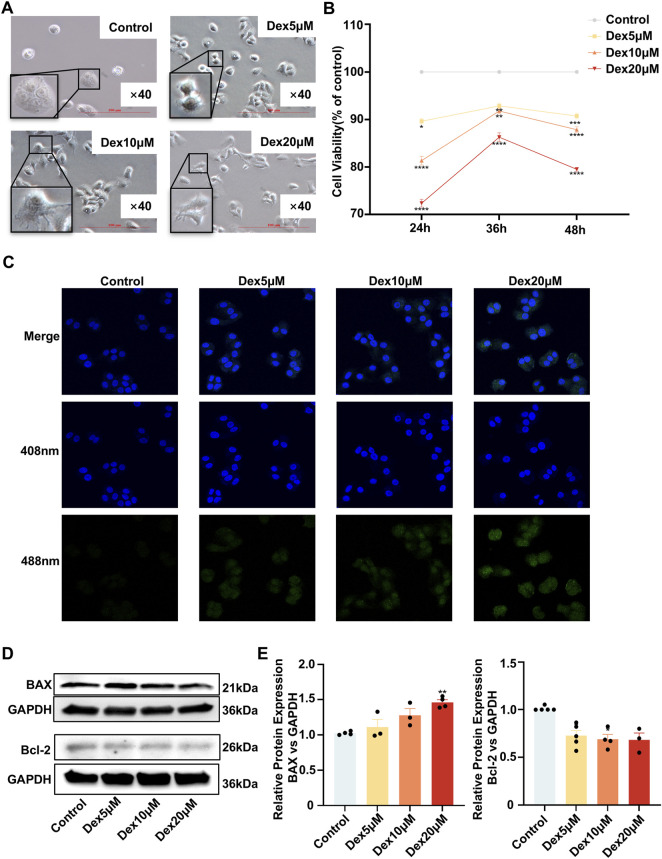
The glucocorticoid dexamethasone decreased the viability and promoted the apoptosis of 16HBE cells. **(A)** Morphological changes in 16HBE cells treated with different concentrations of dexamethasone (microscopy images). **(B)** Compared with control cells, 16HBE cells treated with dexamethasone exhibited decreased viability, as assessed by the CCK-8 assay. **(C)** 16HBE cells treated with dexamethasone exhibited increased apoptosis, as determined by TUNEL staining (green fluorescence). **(D,E)** Protein expression levels of the apoptosis-related markers BAX and BCL-2, as determined by Western blotting **(D)** and quantitative analysis **(E)**. *P < 0.05, **P < 0.01, ***P < 0.001, ****P < 0.0001 using one-way ANOVA.

Under microscopy, the cells in the control group displayed a regular round or oval morphology with clear borders and single nuclei. However, dexamethasone-treated cells demonstrated dose-dependent morphological abnormalities, including irregularity, pleomorphism, and increased bi/multinucleation. These findings indicate that GCs can compromise the normal morphology of airway epithelial cells (Figure 2A).

Concurrently, the results of the CCK-8 assay revealed a dose-dependent decrease in the viability of 16HBE cells with increasing dexamethasone concentrations (Figure 2B), and the TUNEL results revealed that the increase in 16HBE apoptosis paralleled the increase in the dexamethasone dose (Figure 2C). BAX and BCL-2 are critical apoptotic switches that control mitochondrial permeability (Kale et al., 2018). Moreover, Western blot analysis revealed the upregulation of BAX expression in Dex20 μM group (*P* < 0.01) and downregulation of BCL-2 expression in Dex5 μM group and Dex10 μM group (*P < 0.05*) (Figures 2D,E). These results indicate that the GC dexamethasone may decrease cell viability and promote apoptosis in airway epithelial cells.

### 3.4 The GC dexamethasone promoted endoplasmic reticulum stress in airway epithelial cells both *in vitro* and *in vivo*

ER stress, which is activated by the accumulation of misfolded proteins, is primarily mediated through three signaling pathways, i.e., the ATF6-CHOP, IRE1α-XBP1s and PERK-ATF4 pathways.

**FIGURE 3:**
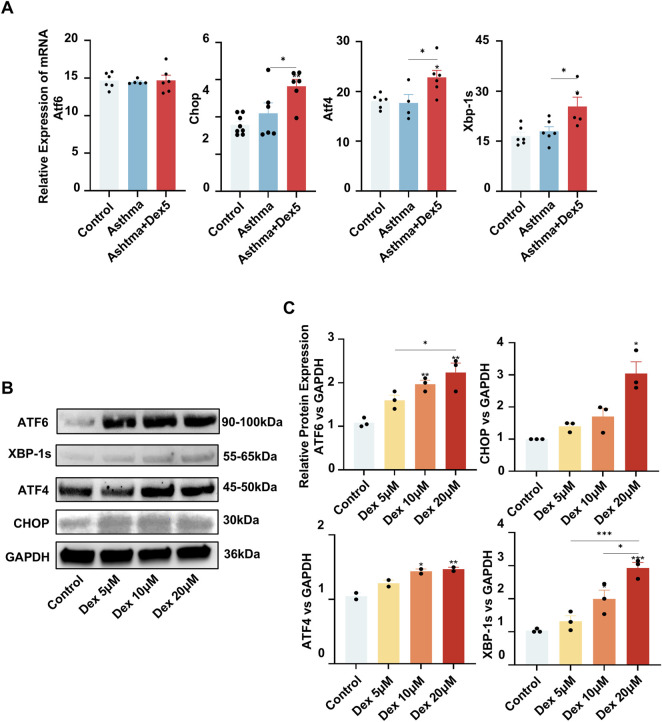
The glucocorticoid dexamethasone promoted endoplasmic reticulum stress in 16HBE cells. **(A)** The expression of several ER stress markers was much greater in the lungs of a mouse asthma model treated with dexamethasone, as determined by RT-qPCR. **(B)** Dexamethasone increased the levels of ER stress markers in the lungs of asthma model mice treated with dexamethasone, as determined by Western blotting. **(C)** Quantitative analysis of the Western blot data shown in **(B)**. *P < 0.05, **P < 0.01, ***P < 0.001 using one-way ANOVA.

To further verify the regulatory effect of GCs on ER stress *in vivo*, we used qPCR to measure the levels of key factors involved in ER stress in mouse lung tissue. Among the control, asthma and asthma + Dex5 groups, the mRNA levels of CHOP were the greatest (*P < 0.05*), and the mean mRNA levels of ATF4 and XBP1 were higher in the asthma + Dex5 group than in the other two groups (Figure 3A).

Compared with the control treatment, dexamethasone dose-dependently increased the protein expression of ATF6, CHOP, ATF4, and XBP1s in 16HBE cells, as determined by Western blotting (*P < 0.05*) (Figures 3B,C).

In summary, the results indicate that the GC dexamethasone may increase ER stress in airway epithelial cells both *in vitro* and *in vivo*.

### 3.5 The GC dexamethasone may have induced apoptosis in 16HBE cells by promoting endoplasmic reticulum stress, and ER stress inhibitors could decrease dexamethasone-induced apoptosis

**FIGURE 4:**
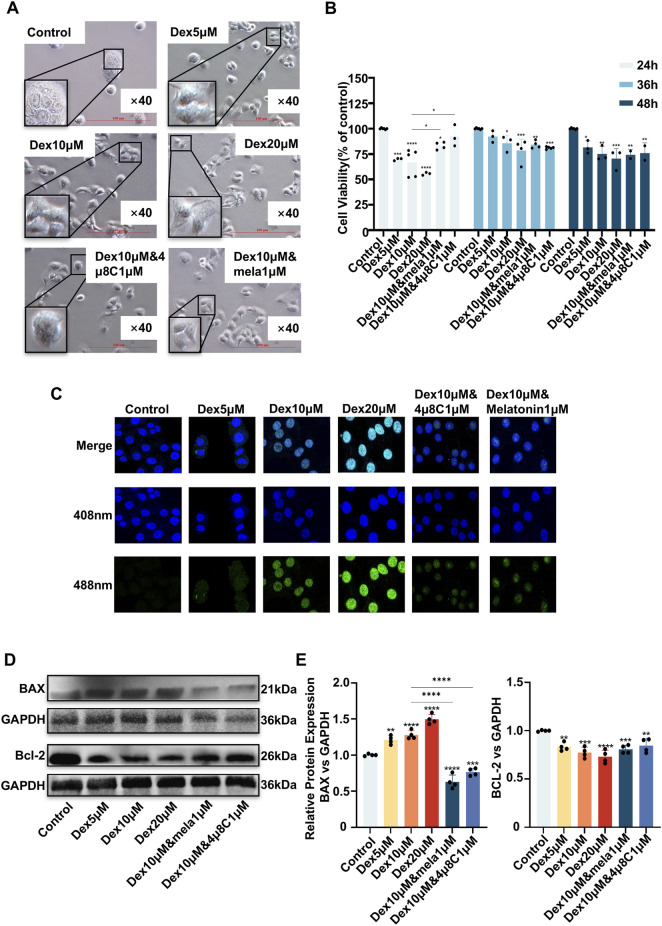
The glucocorticoid dexamethasone induced apoptosis in 16HBE cells by inducing endoplasmic reticulum stress. **(A)** Morphological changes in 16HBE cells treated with different concentrations of dexamethasone (microscopy images). **(B)** Cell viability was assessed by the CCK-8 assay. ER stress inhibitors, including 4μ8C and melatonin, increased the viability of 16HBE cells treated with dexamethasone during the first 24 h. **(C)** ER stress inhibitors decreased apoptosis, as determined by TUNEL staining (green fluorescence). **(D)** ER stress inhibitors decreased the expression of ER stress markers in 16HBE cells treated with dexamethasone, as determined via Western blotting. **(E)** Quantitative analysis of the Western blot data shown in **(D)**. The ER stress inhibitors 4μ8C and melatonin were used. *P < 0.05, **P < 0.01, ***P < 0.001, ****P < 0.0001 using one-way ANOVA.

Compared with the control treatment, dexamethasone induced significant morphological abnormalities in 16HBE cells. These changes were partially reversed by cotreatment with the ER stress inhibitors 4μ8C and melatonin (Figure 4A). Concurrently, 4μ8C and melatonin attenuated the dexamethasone-induced reduction in cell viability, as measured by the CCK-8 assay (*P < 0.05*) (Figure 4B).

Moreover, the TUNEL assay revealed that apoptosis increased in parallel with increased dexamethasone concentrations; additionally, 4μ8C and melatonin seemingly decreased dexamethasone-induced apoptosis in 16HBE cells (Figure 4C).

Furthermore, Western blot analysis demonstrated that these inhibitors alleviated the dexamethasone-induced dysregulation of BAX/BCL-2 expression (*P < 0.05*) (Figures 4D,E).

Collectively, these findings indicate that ER stress inhibitors may decrease GC-induced apoptosis in airway epithelial cells.

## 4 Discussion

Although GCs are the cornerstone of asthma management due to their potent anti-inflammatory effects (Kim and Agrawal, 2025; Niu et al., 2016), they primarily control symptoms rather than providing a cure, and do not provide benefits for all pediatric patients. Approximately 5%–10% of children with asthma exhibit insensitivity to GCs (Reddel et al., 2022). Moreover, in some children, an inadequate response to GCs may even progress to GC-resistant asthma, consequently increasing the risk of acute exacerbation, recurrent hospitalization, and mortality (Tian et al., 2008; Ramos-Ramírez and Tliba, 2022). Our previous study revealed the potential negative effects of GCs on the airway epithelial barrier (Niu et al., 2016). This therapeutic paradox has drawn our attention and motivated further investigation into its underlying mechanisms.

This study revealed that dexamethasone, a GC, could decrease the viability of airway epithelial cells, promote ER stress, and even lead to apoptosis, possibly delaying the repair of the airway epithelial barrier. The ER stress response is a critical pathway activated by various cellular stressors, and we observed that dexamethasone increased the activity of three signaling pathways involved in ER stress, i.e., the ATF6-CHOP, IRE1-XBP1s, and PERK-ATF4 pathways. Activation of these pathways was accompanied by increased expression of pro-apoptotic factors such as BAX and decreased expression of anti-apoptotic factors such as BCL-2, leading to subsequent apoptosis in airway epithelial cells. A decrease in cell viability is induced by several stresses experienced by cells within a bioreactor. This may help to explain why some children with asthma do not respond well to GCs. Meanwhile, we observed that the magnitude of ER stress activation correlated with dexamethasone concentration, suggesting a dose-dependent effect.

In mice, airway stem cells are more abundant in the trachea than in the distal lung (Rock et al., 2010). This regional heterogeneity may cause the tracheal epithelium to be more susceptible to GCs-induced injury, as these cells are actively involved in epithelial maintenance and repair. The concentration of GCs is an important determinant of their negative effects on airway epithelial cells. In human airways, the concentration of GCs is influenced by the volume of periciliary fluid and the GC dose. The average depth of the periciliary fluid layer is approximately 5–45 μM (Fishman, 1977; Sedaghat et al., 2016), and the total volume in the airways of humans is approximately 1 mL–10 mL (Dorscheid et al., 2001; Londino et al., 2017). Inhalation of 200 μg of GCs, assuming 10%–30% deposition in the central airway (Dorscheid et al., 2001), results in a concentration in the sol layer between 2 μM and 100 μM. Moreover, inhaled corticosteroids are often unevenly distributed in the airway, resulting in excessive accumulation of inhaled corticosteroids on part of the airway epithelium barrier. Under conditions of high local GC accumulation, ER stress may be exacerbated, compromising epithelial integrity and impairing barrier function (Wang et al., 2025).

Similar to most other scholars (Osorio et al., 2013; Oh et al., 2002; Rimmer et al., 2021; Kutsuzawa et al., 2023), we believe that inhaled GCs can control asthma in most cases and that their side effects on the airway epithelial barrier can be ignored. However, some stressors, such as airway infection, airway allergen exposure, nutritional element deficiency (Niu et al., 2016), and even mood disorders, can weaken the fragile airway epithelial barrier in patients with asthma and promote ER stress in airway epithelial cells. These stressors may facilitate or even amplify ER stress caused by GCs, leading to or even exacerbating injury to the airway epithelial barrier.

The airway epithelial barrier plays a central role in asthma, as injury to this barrier is a universal pathological feature of the disease (Reddel et al., 2022); on this basis, we propose a mechanistic hypothesis. Specifically, we hypothesize that despite the anti-inflammatory effects and epithelial protective effects at appropriate concentrations of GCs, locally high concentrations of GCs caused by improper inhalation may decrease epithelial cell viability and excessively activate ER stress, causing cells to be in a fragile state and compromising their homeostatic capacity. Concurrently, ER stress may decrease cell viability or even induce excessive epithelial apoptosis, further weakening the airway epithelial barrier. These negative effects of GCs, which are dose dependent, may ultimately diminish the ability of the lung to defend against exogenous insults. The clinical implications of our findings are twofold. First, they highlight a therapeutic paradox: while GCs effectively suppress airway inflammation, they may simultaneously impair the integrity of the airway epithelium, particularly under high local concentrations. This could create a vicious cycle wherein epithelial impairment leads to increased allergen and pathogen insults, exacerbating epithelial inflammation and thus requiring higher GC doses, thereby exacerbating the impairment of airway epithelium. This may be one reason for the incomplete response to long-term inhaled GCs observed in a subset of asthmatic patients. Second, our results suggest that agents aimed at inhibiting ER stress could enhance the therapeutic efficacy of GCs. For example, combining GCs with ER stress inhibitors or agents that promote epithelial repair (e.g., growth factors, retinoids) might preserve barrier integrity while maintaining anti-inflammatory efficacy (Salvi et al., 2022; Peters and Wenzel, 2020; Zhang et al., 2025).

Interestingly, the epithelial injury and apoptosis induced by glucocorticoids, as observed in our study, may have broader implications considering recent advances about asthma pathogenesis. Blocking or slowing down airway remodeling has become a hot topic on asthma (Huang and Pansare, 2019; Propp and Becker, 2013), recent studies has confirmed that the airway remodeling process in asthma is driven by several signaling pathways, including TNFSF11/TGFβ1/STAT3, TGFβ1/Smad3 and TL1A/DR3 axes (Zhang et al., 2021; Zhang et al., 2024a; Zhang et al., 2022; Zhang et al., 2024b). It is known that abnormal proliferation or fibrosis may occur during the repair process when the epithelial barrier is impaired. This suggests that it is highly likely that ER stress-induced apoptosis caused by glucocorticoids may also exacerbate the activation of these pro-remodeling signal pathways.

Although this study reveals potential negative effects of GCs on the airway epithelium, it is important to note that these conclusions are primarily based on cell and animal experiments, and sufficient clinical data are lacking. Meanwhile, the concentrations of dexamethasone used *in vitro* may not precisely reflect those achieved in the airways of patients, although we attempted to model physiologically relevant ranges. Consequently, future *in vivo* studies are warranted to substantiate these findings. Additionally, the development of personalized asthma management strategies to maintain airway epithelial barrier integrity may represent a promising direction for further investigation.

Asthma is among the most prevalent chronic respiratory diseases worldwide, imposing a substantial burden on healthcare systems and contributing to both individual medical expenses and reduced quality of life. Consequently, achieving long-term control of asthma remains a major clinical and socioeconomic challenge. This study proposes a novel mechanism by which GCs may impair the airway epithelial barrier via ER stress-related apoptosis, thereby potentially contributing to GC resistance in patients with asthma. Our findings provide a theoretical foundation for elucidating the mechanisms of GC resistance and for developing improved clinical management strategies for asthma.

## Data Availability

The original contributions presented in the study are included in the article/Supplementary Material, further inquiries can be directed to the corresponding author.

## Supplementary material

SUPPLEMENTARY FIGURE S1 Toxicity of dexamethasone and ER stress inhibitor in 16HBE cells.

SUPPLEMENTARY FIGURE S2 A mouse model of ovalbumin-induced asthma was successfully established.

SUPPLEMENTARY FIGURE S3 Establishment and grouping of the ovalbumin-induced asthma mouse models.
