# Supplementary figures and images for: Glucocorticoids injure the airway epithelial barrier via endoplasmic reticulum stress-related apoptosis in asthma
Source: Front Pharmacol. 2026 May 8;17:1771751. doi: 10.3389/fphar.2026.1771751 (PMC13193804; doi:10.3389/fphar.2026.1771751)

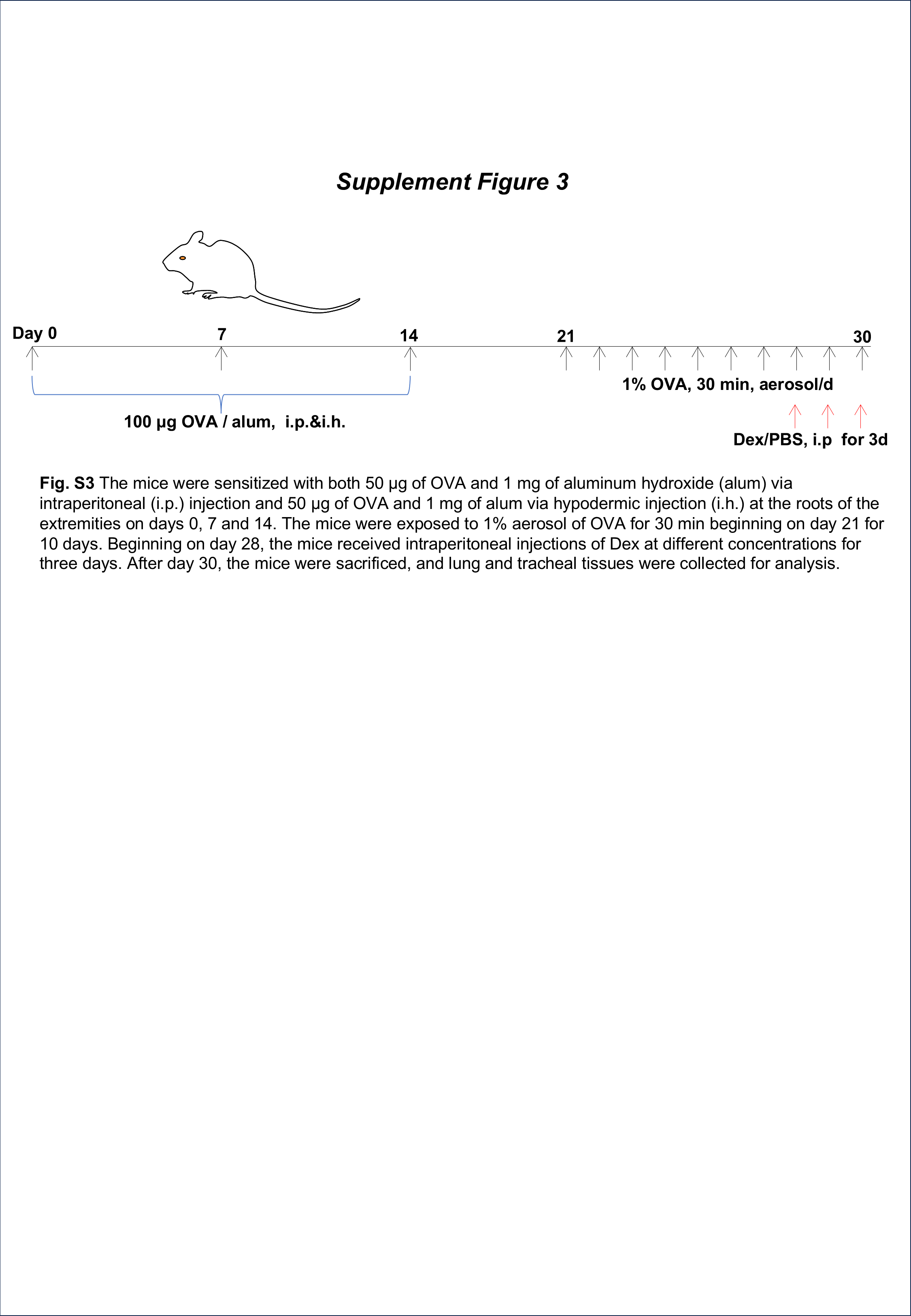

Supplement: Supplementary file 1 [file Image3.tif]

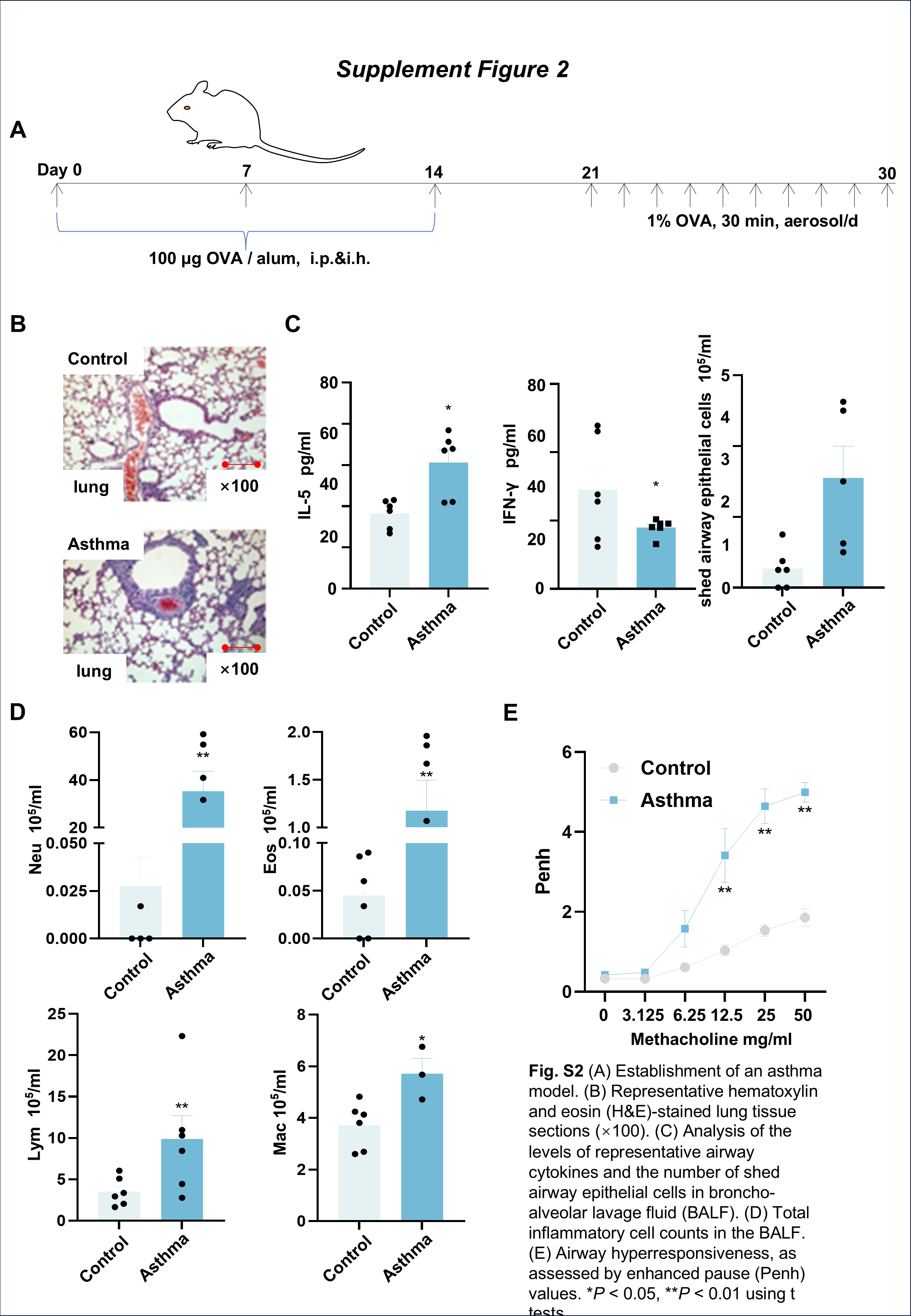

Supplement: Supplementary file 2 [file Image2.tif]

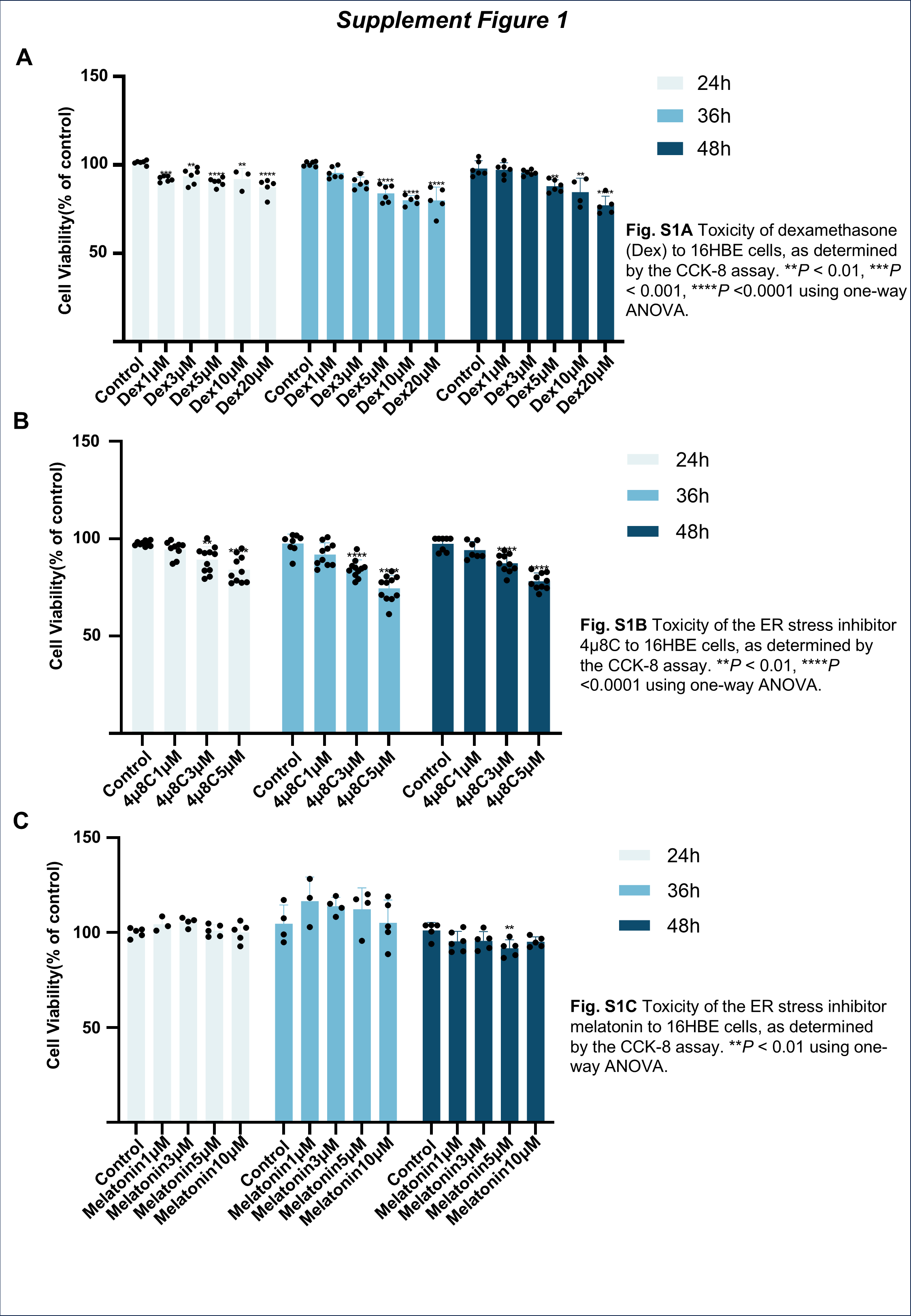

Supplement: Supplementary file 3 [file Image1.tif]
